# Antibiotic Shortages: Pharmacists’ Experiences, Perspectives, and Perceived Clinical Impacts

**DOI:** 10.3390/antibiotics15080808

**Published:** 2026-08-19

**Authors:** Maarten Lambert, Chloé Corrie Hans Smit, Katja Taxis, Lisa Pont

**Affiliations:** 1 Division of Pharmacoepidemiology and Clinical Pharmacology, Utrecht Institute for Pharmaceutical Sciences, Faculty of Science, Utrecht University, Universiteitsweg 99, 3584 CG Utrecht, The Netherlands; 2 School of Clinical and Health Sciences, University of Technology Sydney, Sydney, NSW 2000, Australia; 3 Unit of Pharmacotherapy, -Epidemiology and -Economics, Groningen Research Institute of Pharmacy, Faculty of Science and Engineering, University of Groningen, 9713 AV Groningen, The Netherlands

**Keywords:** drug resistance, microbial, community pharmacy services, pharmacy service, hospital, anti-bacterial agents and drug shortages

## Abstract

**Background**: Australia has experienced high rates of antibiotic shortages, yet little is known about how Australian pharmacists experience and manage them. This study aimed to explore Australian community and hospital pharmacists’ experiences of antibiotic shortages, including their perceived clinical impact and approaches to managing shortages. **Methods**: An exploratory qualitative study using semi-structured interviews was conducted with Australian community and hospital pharmacists between May and August 2024. Participants were recruited via convenience sampling through professional networks and social media. Interviews were audio-recorded, transcribed verbatim, and analysed thematically using an inductive–deductive approach, with coding performed independently by two researchers. **Results**: Fifteen interviews were conducted before data saturation was reached. Sixty percent of participants were female, 67% worked in hospital settings, and pharmacists were located across five Australian states. Four overarching themes were identified: factors driving shortages, communication and collaboration, impact of shortages, and mitigation and prevention. Pharmacists described antibiotic shortages as frequent and unpredictable, attributing them to supply-chain vulnerabilities, limited manufacturing, and market competition. Shortages were reported to affect treatment choice, contribute to antimicrobial resistance concerns, increase pharmacist workload and stress, and negatively affect patients. Pharmacists used strategies such as stock management, collaboration, and importing alternatives, but felt that lasting solutions required coordinated national action. **Conclusions**: Australian pharmacists perceive antibiotic shortages as a complex challenge affecting patient care, antimicrobial stewardship, workload, and healthcare efficiency. While pharmacists have developed reactive strategies to manage shortages, stronger communication, collaboration, and national policy coordination, alongside improved supply-chain resilience, are needed to reduce the impact of future shortages.

## 1. Introduction

Drug shortages have become increasingly common, with an increase of 60% between 2017 and 2019 reported across a sample of 14 OECD countries [1]. Although a spike in drug shortages was seen during the COVID-19 pandemic, followed by a decline in the years after, since 2023 the number of active ingredients in shortage has increased again [2]. Between 2018 and 2023, in the United States, shortages were reported for 258 unique active ingredients [2].

Antibiotic shortages have become a critical issue impacting nations across various income levels and health systems [3]. Pharmacists in the United States and Europe have reported antibiotics as the drugs most often in shortage; annually, hundreds of shortages occur [4,5]. Australia experiences the same pattern, with particularly high rates of antibiotic shortages. Of all oral antibiotics reimbursed in Australia, 86% were affected by at least one shortage in 2022 or 2023 [6]. Reasons proposed for antibiotic shortages include low profitability, complex manufacturing requirements, and market consolidation [7].

### 1.1. Impact of Antibiotic Shortages

Antibiotic shortages can result in treatment interruptions, increased medication errors, and higher healthcare costs [5,8]. Clinicians report that the unpredictability of shortages, limited information, and the lack of suitable alternatives amplify their impact [4,9]. Pharmacists and prescribers have reported increased workload, stress, and frustration as important consequences of shortages [4,10]. Compared to other medications, the implications of antibiotic shortages may be particularly significant, as they are often used for acute treatment. Moreover, antibiotic shortages have been associated with increased use of alternative broad-spectrum antibiotics, potentially contributing to increasing antibiotic resistance rates [8].

### 1.2. Pharmacists’ Role in Handling Antibiotic Shortages

Pharmacists are the healthcare professionals most directly affected by antibiotic shortages and may play a key role in mitigating their impact. Unlike many other medicines, switching antibiotics requires consideration of the spectrum of activity, patient allergies, and antimicrobial stewardship principles [7]. When a shortage occurs, switching to alternatives or substitutes is a frequently used intervention by pharmacists. Switching may be to different brands or generic options, or to another active ingredient [7]. Other reported mitigation strategies include switching to other routes of administration, modifying compounding methods and prioritising specific patient groups [7].

Given the large and increasing number of shortages and the time needed for mitigation strategies, it is not surprising that pharmacists report significant disruptions to their clinical practice [11]. Pharmacists in Austria, Germany, and Kosovo reported that shortages affected up to half of patient encounters and required substantial time to resolve [11]. Besides the increased workload, feelings of frustration and anger were mentioned as impactful for pharmacists, with excessive regulation and bureaucratic obstacles as important barriers to more efficient mitigation of shortages [11]. Although previous studies have described pharmacists’ experiences with drug shortages in Europe and North America [4,5], little is known about how Australian pharmacists experience and manage antibiotic shortages specifically. Therefore, this study aimed to explore Australian community and hospital pharmacists’ experiences of antibiotic shortages, including their perceived clinical impact and approaches to managing shortages.

## 2. Methods

### 2.1. Study Design and Setting

An exploratory qualitative semi-structured interview study was performed from May–August 2024 in Australia. The study followed the consolidated criteria for reporting qualitative studies (COREQ) checklist [12].

### 2.2. Target Population and Recruitment

Potential participants were recruited through convenience sampling via professional body membership networks and social media. Community and hospital pharmacists involved in dispensing antibiotics and practising in any Australian context were eligible for inclusion. Written informed consent was obtained from all participants. Interviews continued until data saturation was reached, operationalised as no new themes emerging from three consecutive interviews. Pharmacists participated voluntarily and were not compensated for their time.

### 2.3. Interviews

The interviews were conducted in person or via video conference and a predefined semi-structured interview guide was used (Appendix A). The interview guide was developed based on a review of the existing literature on antibiotic shortages, with particular attention to studies describing drivers, clinical impact, and management strategies for antibiotic shortages. The guide was developed by M.L. and discussed and reviewed by M.L. and L.P. The interview guide was not pilot tested and was designed for an interview duration of approximately 10–15 min to minimise participant burden and facilitate participation by pharmacists within busy clinical work environments, while allowing for sufficient depth in answering. The interviews were conducted one-on-one by M.L. while pharmacists were in their work environment, with measures taken to ensure a private conversation. Prompts were provided by the interviewer when initial responses lacked detail. As data saturation may be reached with up to fifteen interviews [13], we anticipated conducting at least fifteen interviews and looked for data saturation following all interviews after interview nine. All interviews were audio-recorded.

### 2.4. Data Analysis

Audio-recordings were transcribed verbatim using Microsoft Word Transcribing 365 (Microsoft Corporation, Redmond, WA, USA). All transcriptions were manually checked by M.L. and anonymised on the same day of the interview. Recordings were not stored by the transcribing software and were destroyed after transcription. The transcripts were not returned to participants for comment. Data were thematically analysed using NVivo 14 Software (Lumivero, Denver, CO, USA), following an iterative inductive–deductive approach.

A pragmatic inductive–deductive thematic analysis was utilised. Coding was primarily data-driven, while the interview guide and study objectives informed attention to pre-specified areas such as perceived causes, clinical impact, communication and mitigation strategies. Emerging codes and preliminary themes were discussed iteratively between two researchers (M.L. and C.C.H.S.) throughout the analysis process. Both researchers independently reviewed and coded the full dataset. Codes were then compared, refined, and grouped into overarching themes and subthemes through discussion with K.T. and L.P. [14].

### 2.5. Ethics

This study was approved by the University of Technology Sydney (UTS) Human Research Ethics Committee (UTS HREC REF NO. ETH24-9102).

### 2.6. Research Team and Reflexivity [12,15]

All interviews were conducted and transcribed by M.L. (male, PharmD, PhD) and independently coded and analysed by M.L. and C.C.H.S. (female, PharmD, PhD). M.L. holds a PhD in antibiotic use, antibiotic resistance, and pharmacy practice, with a specific focus on the community setting. C.C.H.S. holds a PhD in understanding antibiotic resistance in the community and associated risk factors including antibiotic use. Neither researcher had a prior established relationship with the participants. At the time of the research, both were full-time researchers and university teachers. M.L. did not have clinical experience, whereas C.C.H.S. had clinical experience in Australian community pharmacy practice. L.P. is a registered pharmacist in Australia and a professor in the Discipline of Pharmacy. She has extensive experience in clinical pharmacy and quality use of medicines research, including drug utilisation and qualitative research methods. KT is a registered pharmacist in Germany and a professor of pharmacotherapy and clinical pharmacy in the Netherlands with extensive research experience in qualitative research.

Researcher characteristics may have influenced the development of the interview guide and the interpretation of the participants’ perspectives. Researchers without clinical experience may create an outsider position with regard to clinical practice, while those with clinical experience ensure that clinically relevant topics are covered in the interview. The researchers’ prior research on and experience with antibiotic shortages may have shaped the researchers’ expectations and sensitivity to particular themes during data analysis. To mitigate these influences, coding and thematic analysis were conducted independently by two researchers, working from fully anonymised transcripts.

## 3. Results

### 3.1. Participants

A total of fifteen interviews were conducted, at which point data saturation was reached. There were no dropouts or withdrawals from the study after the signing of informed consent forms. Data on refusal to participate could not be collected. All interviews had a duration of 11–20 min. Of the fifteen pharmacists, nine pharmacists (60%) were female, ten (67%) were hospital pharmacists and five (33%) practised in the community setting. The pharmacists were located in five different states: New South Wales (*n* = 5), Western Australia (*n* = 5), Queensland (*n* = 2), South Australia (*n* = 2), and Victoria (*n* = 1).

### 3.2. Experience with Shortages

All pharmacists had experience with antibiotic shortages. There was some variation to the perceived extent of shortages, but generally, pharmacists across Australia described antibiotic shortages as a frequent and evolving challenge, with a general perception that the occurrence of shortages had increased over time, in particular since the COVID-19 pandemic. Respondents characterised shortages as ongoing or recurrent, with some noting that they have become more frequent and prolonged, although others mentioned slight improvements in 2024.

A wide range of antibiotics were mentioned as affected by shortages, including commonly used agents such as amoxicillin, cefalexin, azithromycin, clarithromycin, and trimethoprim, as well as intravenous therapies and special access or imported medications like pristinamycin which are used in specialised acute care settings. Shortages were described as highly variable and difficult to predict, with no clear pattern in their timing, duration, or the specific medicines affected.

*I feel like it’s increasing (…). Antibiotics (…), I mean just in the case of amoxicillin or cefalexin, you know, they’re such ubiquitous medications that we really felt that.*—**Pharmacist 7**

*But I think for antibiotics in the last 12 months, they haven’t been as big a deal. There’s a couple that have sort of popped their head up and we’ve monitored that generally we’ve had access to stock.*—**Pharmacist 2**

### 3.3. Pharmacists’ Perspectives on Antibiotic Shortages

Four overarching themes on the pharmacist perspective emerged from the interviews: (1) factors driving shortages, (2) communication and collaboration, (3) impact of shortages, and (4) mitigation and prevention of shortages. Each theme comprised several subthemes (Table 1).

#### 3.3.1. Theme 1: Perceptions of the Factors Contributing to Shortages

The pharmacists perceived multiple, often interconnected factors contributing to antibiotic shortages. A key driver perceived as contributing to shortages was disruption to international supply chains, particularly as Australia relies heavily on imported medicines and has limited local manufacturing capacity. Participants highlighted vulnerabilities such as reliance on a small number of manufacturers or single production sites. Broader logistical challenges, including COVID-related disruptions, transport delays, and geopolitical instability, were also perceived to have ongoing impacts on supply. Economic factors were frequently noted, with Australia considered a less profitable market than many other countries due to pricing structures such as the Pharmaceutical Benefits Scheme, potentially reducing its priority for pharmaceutical companies.

‘*Antibiotics are not really the number one best thing to be investing in terms of research and development (…). So, there has been a general sort of decrease in the antibiotic pipeline driven by profit.*’—**Pharmacist 9**

At a local level, participants described demand-related pressures, including sudden increases in prescribing during outbreaks and subsequent snowballing, which can rapidly exhaust supply and create cascading shortages in alternative agents (e.g., mycoplasma leading to increased azithromycin use). Some respondents noted that supply–demand mismatches, formulary changes, and procurement processes could further negatively impact availability. Stock management practices, such as hoarding or uneven distribution across pharmacies and hospitals, were also seen to exacerbate shortages. Despite these insights, there was also considerable uncertainty, with several pharmacists reporting limited transparency and often not knowing the exact causes of specific shortages.

‘*with the azithromycin shortage, because that went out, people started using clarithromycin and that has subsequently gone out as well (…). So then it has a snowball effect on other antimicrobial shortages.*’—**Pharmacist 1**

Pharmacists also described shortages as increasing competition between pharmacies, with all pharmacies competing for the same available stock. This competition was seen as problematic with participants perceiving it as contributing to hoarding and initiation of new shortages, or exacerbating or extending of existing shortages.

‘*And sometimes one place will buy up all the available stock, which is not great unless it’s us.*’—**Pharmacist 9**

#### 3.3.2. Theme 2: Communication and Collaboration

Australian pharmacists mentioned that information on shortages was obtained from a variety of sources. Informal professional networks complemented formal government and regulatory notification systems and facilitated rapid information exchange and shared decision-making. Variability in communication effectiveness was noted, with challenges including delays, inconsistent information dissemination, and gaps in awareness among stakeholders. In some hospitals, the pharmacists reported that formalised processes such as regular shortage meetings, internal communications, real-time inventory monitoring systems, and updated clinical guidelines, had been implemented based on earlier experience with shortages.

‘*Often times you will be told about the shortage and you have to work collaboratively within the multidisciplinary team to kind of inform the key stakeholders. So our normal process would be, we have weekly shortage meetings, that’s how bad it is*.’—**Pharmacist 8**

‘*It’s through a variety of resources, so word of mouth (…), the TGA website, the company notifications, also procurement, wholesaler identifying, clinicians on the ground (…), it’s really a multi-pronged approach.*’—**Pharmacist 14**

There was a diverse range of views regarding the quality and timing of notifications of antibiotic shortages, and discussion regarding whether notification was the responsibility of national or state-based authorities. Some pharmacists noted that sometimes notifications would only reach them after the shortage had already been resolved, while other pharmacists mentioned that notifications would only reach them once shortages became significant.

*‘(…) sometimes what happens is you hear about the shortage and then by that time, you hear about, it’s no longer an issue.*’—**Pharmacist 3**

‘*So sometimes shortages happen, and by the time we know about it, things are pretty pretty bad*.’—**Pharmacist 1**

While there was conversation of shortages driving competition between pharmacists, the pharmacists also noted that shortages resulted in increased collaboration between pharmacies particularly around identification of suitable alternatives that could be recommended as a replacement for antibiotics experiencing a shortage, as well as collaboration around development of resources on antibiotic shortages for prescribers. Collaboration could be formal, such as a local or state-based network, or informal, relying on personal networks from individual pharmacists.

In the hospital setting, collaboration was perceived to extend beyond a single pharmacy environment with inter-hospital networks and state-based systems described. The pharmacists reported that collaboration needed to also involve wholesalers and international suppliers, particularly when sourcing alternative or unregistered medicines through special access pathways.

‘*You know, sometimes we will directly deal with import or exporters ourselves. Sometimes we go through some people in our pharmacy department can help. It depends what they’re up to. And then sometimes at a state based level, we can convince the state wholesaler to do that leg work.*’—**Pharmacist 3**

In community pharmacy, pharmacists described a similar need for additional communication around shortages, including liaising with prescribers, identifying stock availability across pharmacy networks, and counselling patients. Frustration about regulatory requirements limiting pharmacist options to independently modify formulation and therapies was also noted.

‘*And being a pharmacist in Australia, unfortunately, I cannot even change the formulation, so I can’t give a tablet instead of a liquid. I can’t change anything on the script.*’—**Pharmacist 1**

#### 3.3.3. Theme 3: Perceived Impact of Antibiotic Shortages

Four key subthemes emerged around the perception that shortages had on pharmacy practice. These subthemes were impact on choice of treatment, impact on antimicrobial resistance, and impact on pharmacist workload and economics, and psychosocial impacts.

Perceived impact on choice of treatment was described in the context of treatment effectiveness, adverse effects associated with treatment and contribution to antimicrobial resistance. Pharmacists described that shortages among first-line antibiotics may result in other options being selected with concerns that non-first-line alternatives may not be as effective, potentially resulting in more severe infections, increased burden of side effects, or longer treatment duration or hospitalisation.

‘*I do think for patients, there are changes and there are risks associated with it as well. From a clinical perspective, you’re definitely not giving first line. You’re then opting for second line, third line.*’—**Pharmacist 4**

Interestingly, pharmacists described antibiotic shortages as both potentially contributing to and decreasing antimicrobial resistance. There were concerns that shortages in some antibiotics may lead to increased use of broad-spectrum antibiotics compared to first-line antibiotics, which could lead to increased antimicrobial resistance. Conversely, some pharmacists noted that if the shortage was for a broader spectrum agent, then shortages may decrease the use of a particular antibiotic, therefore decreasing utilisation.

‘*I think it’s something that is a high risk and certainly has a huge impact on healthcare. And as I alluded to, it does impact patient outcomes and it does promote antimicrobial resistance if you don’t have access to first line therapies.*’—**Pharmacist 9**

‘*Sometimes I do find a shortage is a really good thing as well. If I’m trying to actually make people use less of a certain antimicrobial. So it goes both ways, which is kind of funny.*’—**Pharmacist 8**

A second subtheme centred around the impact of antibiotic shortages on pharmacist workload and the associated economic cost from the pharmacist’s perspective. Pharmacists from both the hospital and community settings spoke about an increased workload associated with antibiotic shortages, particularly around rationing. In the hospital setting, antibiotics in shortage may be limited to use within specific populations, which had a considerable perceived cost in terms of pharmacist time in the hospital setting.

‘*Say if we got in something that was a different strength or a different way to prepare, then we would have to change the guidelines. Put it all through the policy process, (…) and then update smart infusion pumps, which again have to be tested after you’ve plugged them in, then approved, then released, and then you have to (…) communicate with your colleagues (…). So, there’s quite a lot of yeah, workload costs.*’—**Pharmacist 7**

In the community setting, rationing was also mentioned. This provided additional time to source supply of an antibiotic, but pharmacists perceived an increased workload and negative impact on patients because of rationing.

‘*So rather than getting (…) 30 tablets, so that was 10 weeks’ worth (…) we give them four weeks’ worth, so they only get 12 tablets.*—**Pharmacist 7**

Economic impact was considered across several areas. Higher costs resulted from increased pharmacist workload, because of rationing as described above or because of increased workload associated with sourcing stock. Importing products from abroad impacted pharmacists’ workload and increased the cost of antibiotics as they can be several times more expensive than first-choice antibiotics.

‘*Waste of time and a waste of money because it’s very costly to, you know, pay us to spend that time doing something we could be doing something better than obviously, all the changes that come with it.*’—**Pharmacist 7**

The pharmacists were also aware of the psychosocial impacts of antibiotic shortages, raising increased stress for patients who may need to go back to a physician to prescribe an alternative antibiotic or who may need to return to the pharmacy multiple times to receive the entire quantity of the prescribed antibiotic. Pharmacists also spoke about the stress that antibiotic shortages cause for them personally.

‘*We were scrambling at the time. It was very stressful as a pharmacist because we knew that the alternatives were not as good at preventing the particular disease that we were trying to treat.*’—**Pharmacist 2**

#### 3.3.4. Theme 4: Mitigation and Prevention

Participants emphasised the need for coordinated action at national and systemic levels. Strengthening collaboration between regulatory bodies, professional organisations, and clinicians was viewed as essential, alongside greater political engagement to prioritise the issue. While there was a recognition that the problem of antibiotic shortages was beyond the control of individual pharmacists, the pharmacists mentioned different approaches to mitigating the impact of shortages related to antibiotics, many of which had evolved over the past years with increased experience with shortages.

‘*I don’t think it’s something that a pharmacist themselves on the ground can resolve, it definitely needs to be escalated in a national way.*’—**Pharmacist 3**

Stock management was a key area where pharmacists spoke about strategies used to ensure adequate antibiotic supply. Examples were provided by pharmacists who would increase stock holdings of specific antibiotics in preparation for a potential shortage, or who would try to increase stock by importing international products as permitted under temporary Australian legislation to address shortages.

‘*to just keep more stock within the hospital to also buffer.*’—**Pharmacist 5**

Pharmacists saw local national manufacturing as an important strategy to prevent future antibiotic shortages. There were different views on the extent to which antibiotics should be manufactured in Australia. Some pharmacists mentioned this should be done for antibiotics in general, while others mentioned local production specifically for certain key antibiotics. In addition to increasing the local supply chain, pharmacists raised the need for a national approach to stockpiling.

‘*I’d obviously like us to see Australia manufacture more of these drugs on their shores as well.*’—**Pharmacist 1**

Further legislation change was also mentioned to give pharmacists more opportunity to change a prescription without referring the patient back to the prescriber, including pharmacist-led changes in dose or formulation such as a change from tablet to liquid as a potential approach to minimise the burden on healthcare professionals and patients.

‘*to be able to manage shortages in a sensible way, without having to bring your doctor for every 3 milligram change in dose.*’—**Pharmacist 3**

## 4. Discussion

This study provides the first qualitative exploration of Australian pharmacists’ experiences with antibiotic shortages, offering insight into how shortages affect clinical practice and identifying opportunities for national policy reform. The key findings of this research are that Australian pharmacists perceive antibiotic shortages as an increasingly common and complex issue. They experience that this negatively affects patient care, antimicrobial stewardship, pharmacist workload, and healthcare efficiency. While pharmacists have developed collaborative strategies to manage shortages, they believe lasting solutions require coordinated national policies, stronger communication, and more resilient medicine supply chains.

### 4.1. International Pharmacists’ Perspectives on Shortages

Although literature specifically identifying pharmacists’ attitudes toward antibiotic shortages is rare, perspectives on shortages in general have been explored to some extent. Community pharmacists in Belgium stressed the time-consuming nature of managing drug shortages, the stress shortages cause for pharmacists and patients, and negative financial aspects nationally and for individual pharmacists [16]. These views largely overlap with those of the Australian pharmacists in this study. From interviews with stakeholders in the United States, including pharmacists, similar themes to those in this study emerged. Inadequate resources and lack of timeliness of notifications on shortages were acknowledged as factors that increase the negative impact of shortages. They advocated for collaborative resources to be used by healthcare professionals, policymakers, and manufacturers [17]. This seems to align with views from pharmacists in Australia who specifically mentioned the benefits of intensive collaboration to minimise the impact of shortages.

The impact of shortages has also been previously described. Clinical, economic, and humanistic consequences have all been attributed to drug shortages in general and to antibiotic shortages specifically [8,18,19,20,21], as is also the case in this study. Similarly, concerning policy changes to mitigate the number or impact of shortages, the results of this study overlap with existing literature. Australian pharmacists stressed the importance of policy reforms to give pharmacists more possibilities to mitigate the impact of shortages. Moreover, improving supply chain resilience and local production were identified as important strategies. Similar strategies have been suggested by pharmacists internationally for drug shortages in general [22].

It is important to consider that perspectives on general drug shortages may deviate from those specific to antibiotics, especially since research showed that different shortages, i.e., long-term versus short-term or different active ingredients, are handled differently and have varying impacts [21,23]. To illustrate, the impact of long-term shortages may diminish as physicians and pharmacists adapt by using alternative therapies. Yet, longer antibiotic shortages can have wider public health consequences by increasing the use of suboptimal broad-spectrum antibiotics and promoting antimicrobial resistance [21]. Moreover, characteristics of individual pharmacies and pharmacists, e.g., size, location and work experience, influence the impact of shortages, as was mentioned in this study and reported earlier [24]. Still, there seems to be consensus on a lot of aspects of shortages between Australian pharmacists and pharmacists internationally.

### 4.2. International Strategies to Mitigate the Impact of Shortages

Consistent with previous international research, the Australian pharmacists in this study perceived that effective responses require coordinated action across the health system rather than reliance on individual practitioners or organisations [8,17,25,26]. A systems view of drug shortages proposes three broad approaches to addressing shortages: allowing market forces to respond, identifying alternative therapies or supply pathways, and introducing regulatory or policy changes to facilitate management of shortages [27]. The findings of this study align most closely with the latter two approaches. Participants highlighted three broad areas for improvement: more timely and transparent communication about shortages, greater flexibility for pharmacists to manage shortages in clinical practice, and policies that strengthen medicine supply-chain resilience. These priorities are consistent with recommendations from international experts, who have emphasised improved coordination and collaboration [17], regulatory adaptation [3], manufacturing transparency [25], and broader policy and system-level approaches [8] as key strategies for mitigating the impact of shortages.

A notable finding of this study was the emphasis placed on national coordination. Pharmacists reported variable access to information regarding shortages and uncertainty about their underlying causes, suggesting that enhanced national surveillance, communication, and planning mechanisms may support more proactive management of shortages. Similar calls for improved coordination and communication have been made internationally [8,17,25]. Participants also advocated measures such as increased strategic stock holdings, selective local manufacturing capacity for essential antibiotics, and regulatory reforms that would enable pharmacists to implement clinically appropriate substitutions when shortages occur. Similar approaches have been proposed internationally, including strengthening security stocks [27], increasing domestic manufacturing capability [28], diversifying supply sources [22], and introducing regulatory measures to facilitate management of shortages [25,28].

### 4.3. Implications for Antimicrobial Stewardship Practice

Shortages and shortage-driven substitution can have direct implications for antimicrobial stewardship, and antimicrobial stewardship teams can support effective antibiotic use, including during shortages [29]. When first-line agents become unavailable, pharmacists and prescribers may be forced to use broader-spectrum or otherwise less optimal alternatives, which can conflict with guideline-concordant prescribing and local antimicrobial stewardship policies. Because pharmacists in this study described relying predominantly on reactive strategies, such as stock buffering and ad hoc importing, once a shortage had already emerged, more proactive, system-level tools could help translate these reactive practices into standardised, anticipatory responses. Real-time shortage-reporting platforms linked to wholesaler and manufacturer data could give pharmacists earlier warning than the informal, word-of-mouth networks described by participants. Integrated stock-surveillance systems spanning hospital and community sectors could reduce the uneven distribution and hoarding behaviours pharmacists described, while predictive analytics applied to procurement and prescribing data may help anticipate emerging shortages. Artificial intelligence could be used to support these tools, as a 2025 meta-analysis reports that machine learning models offer promising enhancements to traditional stewardship strategies [30].

### 4.4. Strengths and Limitations

An important strength of this study is that it is the first to analyse pharmacists’ perspectives on antibiotic shortages specifically. This is important as antibiotics are the drug class most often affected by shortages and their impact can be severe. Other strengths of the study include recruiting pharmacists in hospitals and the community from different parts of Australia, and conducting interviews until data saturation, which ensures a comprehensive and complete mapping of views and attitudes.

There are also limitations, some of which are inherent to conducting qualitative research. Researchers and participants can be affected by several forms of bias that may influence the results, including but not limited to interviewer bias, social desirability bias, volunteer bias, and recall bias. Our sample was skewed towards hospital pharmacists (67%), reflecting the convenience sampling strategy used to recruit participants through professional networks. This may have shaped the findings, as hospital and community pharmacists can experience and manage antibiotic shortages differently: hospital pharmacists typically operate within multidisciplinary teams and centralised procurement systems, whereas community pharmacists more often manage shortages independently and have more direct, ongoing contact with patients. Consequently, perspectives specific to community pharmacy practice, such as the financial impact on individual pharmacy owners or the burden of repeated patient consultations, may be underrepresented in our findings. Furthermore, the interviews in this study were conducted during work hours and designed to be relatively short. In practice, the duration of the interviews was not so strict; in some interviews, pharmacists shared their perspectives within just over ten minutes, while in others pharmacists were interviewed for close to twenty minutes to gather as much information as possible. Yet, additional detail on some perspectives through longer interviews could have benefited the depth of the analysis. Extrapolation of these results to other countries is challenging because of great international variations in definitions of shortages and policies regarding drug shortages [29,31].

## 5. Conclusions

Australian pharmacists perceive antibiotic shortages as an increasingly frequent and complex challenge with significant consequences for patient care, antimicrobial stewardship, pharmacist workload, and healthcare systems. While pharmacists have developed practical strategies to manage shortages, these are largely reactive and cannot address the underlying drivers. Strengthening communication, collaboration, and national coordination, alongside policies that improve supply chain resilience and support local manufacturing, may help reduce the impact of future shortages. Further research should evaluate the effectiveness of these strategies and inform evidence-based policies to improve preparedness for antibiotic shortages.

## Figures and Tables

**Table 1:** Themes and subthemes identified and key findings per subtheme.

Theme 1: Perceptions of the Factors Contributing to Shortages	Key Findings
Infection outbreaks and cascading shortages	Sudden demand spikes during outbreaks rapidly depleted stock and triggered secondary shortages of alternative antibiotics.
Limited manufacturers	Reliance on few manufacturers or single production sites was seen as an important vulnerability
Competition between pharmacies for stock	Pharmacies competing for the same limited stock was perceived to trigger or worsen shortages through hoarding behaviour.
Theme 2: Communication and Collaboration	
Notification of shortages	Information came from multiple, inconsistent sources. Formal notifications often arrived late, after a shortage had already resolved or become severe.
Collaboration and competition between pharmacies	Shortages resulted in both collaboration (sharing stock or information) and competition for supply, across pharmacies, hospitals, and sectors.
Theme 3: Perceived Impact of Antibiotic Shortages	
Treatment choice	Shortages of first-line agents forced use of non-first-line alternatives, raising concerns about reduced effectiveness and greater side-effect burden.
Antimicrobial resistance	Shortages were perceived to both increase and decrease resistance risk, depending on which agent was affected.
Pharmacist workload and economics	Managing shortages (sourcing stock, rationing, updating guidelines) increased workload and costs in community and hospital pharmacies.
Psychosocial impacts	Shortages caused stress for pharmacists and inconvenience for patients needing repeat pharmacy visits or prescriber referral.
Theme 4: Mitigation and Prevention	
Coordinated national and systemic actions	Pharmacists called for stronger national coordination, greater political engagement, and closer collaboration between regulators, professional bodies, and clinicians.
Stock holdings	Increasing buffer stock and importing alternatives under temporary provisions were commonly mentioned reactive strategies.
Legislation change	Pharmacists wanted expanded legal authority to substitute dose or formulation without prescriber referral.

## Data Availability

The data presented in this study are available on request from the corresponding author due to ethical and privacy reasons.

## Appendix A. Interview Guide

As mentioned on the consent form, this interview group will be audio-recorded for transcription purposes. The transcription will be deidentified and, once the transcripts have been created and verified, the audio recordings will be destroyed.

Do you have any questions before we start?

(1) Demographics:
  ○ What is your Profession
  ○ Do you work in the hospital or community setting
  ○ What is your role in medicines management
    ■ Prompts:
      - prescribing
      - supply
(2) What experience have you had with antibiotic shortages over the past 12 months?
  ○ Prompt: any particular medicines?
(3) How do you think antibiotic shortages have impacted patient care?
  Prompts:
  ○ what about the choice of therapy
  ○ what about your clinical goals
  ○ what about quality of medicine use
  ○ what about clinical workload such as increased time and workload
(4) What do you think the impact of medicines shortages has been for you patients?
  ○ What about access to medicines?
  ○ What about timeliness of care?
  ○ What about cost of medicines?
(5) How have you dealt with any antibiotic shortages you have experienced
  ○ alternate medicine
  ○ alternate non-drug therapy
  ○ alternate dose
  ○ no treatment
(6) What strategies do you think could be used to minimise the impact of antibiotic shortages on patient care?
  ○ better notification of shortages
    ■ explore awareness of/ use the TGA medicines shortages database
  ○ clinical guidance regarding alternative when a shortage occurs
  ○ collaboration between health professionals
  ○ anything else?
